# Colon adenocarcinoma and Birt–Hogg–Dubé syndrome in a young patient: case report and exploration of pathologic implications

**DOI:** 10.1080/15384047.2023.2184153

**Published:** 2023-03-01

**Authors:** Grant W. Jirka, Daniel S. Lefler, Jessica Russo, Babar Bashir

**Affiliations:** a Department of Internal Medicine, Thomas Jefferson University, Philadelphia, PA, USA; b Department of Medical Oncology, Sidney Kimmel Cancer Center, Thomas Jefferson University, Philadelphia, PA, USA; c Guardant Health Inc, Redwood City, CA, USA; d Department of Pharmacology & Experimental Therapeutics, Thomas Jefferson University, Philadelphia, PA, USA

**Keywords:** Birt–Hogg–Dubé syndrome, colorectal cancer, folliculin gene, FLCN mutation, Wnt signaling

## Abstract

Birt–Hogg–Dubé syndrome (BHD) is an autosomal dominant disorder caused by germline mutations in the folliculin gene (*FLCN*) that result in the functional loss of the tumor suppressor folliculin. It is classically associated with cutaneous hamartomas, pulmonary cysts with spontaneous pneumothorax, and various renal cancers. In this case, we present a patient initially diagnosed with chromophobe renal cell carcinoma and subsequently found to have colorectal cancer (CRC). The presence of two separate malignancies in a young patient with a strong family history of CRC (father and paternal grandfather) led to genetic testing, which revealed an *FLCN* c.1177–5_1177-3del mutation, and a diagnosis of BHD was made. Out of the more than 300 known unique mutations of the *FLCN* coding region, the c.1285dupC mutation on exon 11 has been the only one convincingly associated with CRC thus far. While larger cohort studies are needed to further clarify this association, we present the first patient with CRC to our knowledge with an *FLCN* c.1177–5_1177-3del mutation and loss of heterozygosity implicating it as an initiating factor in tumorigenesis. We further explore the studies supporting and refuting the connection between BHD and CRC and highlight the molecular signaling pathways that may play a role in pathogenesis.

## Case presentation

**Figure 1.**
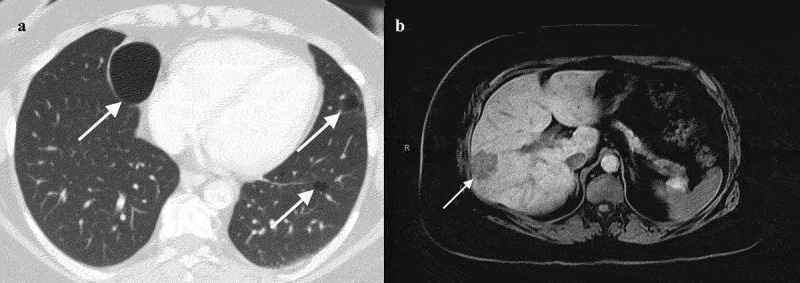
Clinical imaging.

Note: (a) Bilateral pulmonary cysts (arrows) at diagnosis, largest measuring 5.3 × 4.1 cm along the right heart border, (b) Solitary liver lesion (arrow) in segment 5 discovered at disease recurrence and measured 2.6 × 1.9 cm.

A 47-y-old Caucasian woman with a history of resected chromophobe renal cell carcinoma (RCC) presented to our oncology clinic after being diagnosed with rectosigmoid colon adenocarcinoma on routine screening colonoscopy. She was found to have an 8 cm partially circumferential tumor in the distal sigmoid colon that was microsatellite stable (MSS), and molecular tests for *BRAF, KRAS*, and *NRAS* mutations were negative. Abdominal imaging obtained for evaluation of her RCC 4 months earlier did not detect this mass, but subsequent computed tomography (CT) confirmed the colonoscopic findings of a luminal lesion without enlarged regional lymph nodes or distant disease. Imaging evaluation also revealed bilateral pulmonary cysts (Figure 1a), though she had no personal history of spontaneous pneumothorax.

She underwent a rectosigmoid resection, and pathology evaluation revealed a stage IIA (pT3N0) moderately differentiated adenocarcinoma based on evaluation of 18 regional lymph nodes. As no high-risk features were present, she was not offered adjuvant chemotherapy. However, given the finding of two primary malignancies in a relatively young patient along with bilateral pulmonary cysts, she was referred to our institution’s genetic counseling team. Comprehensive next-generation sequencing (NGS) multigene panel testing done via saliva identified the presence of a pathogenic germline mutation in the folliculin (*FLCN*) gene, c.1177–5_1177-3del (intronic) on exon 11 (Supplementary Table 1). Thus, she was diagnosed with Birt–Hogg–Dubé syndrome (BHD). Given her new diagnosis and its attendant risk of pneumonthoraces, she was referred to a pulmonologist who elected to pursue clinical monitoring and serial CT scans. She also was further evaluated by dermatology and was found to have multiple benign hamartomas that were clinically determined to be fibrofolliculomas, consistent with BHD.

**Figure 2.**
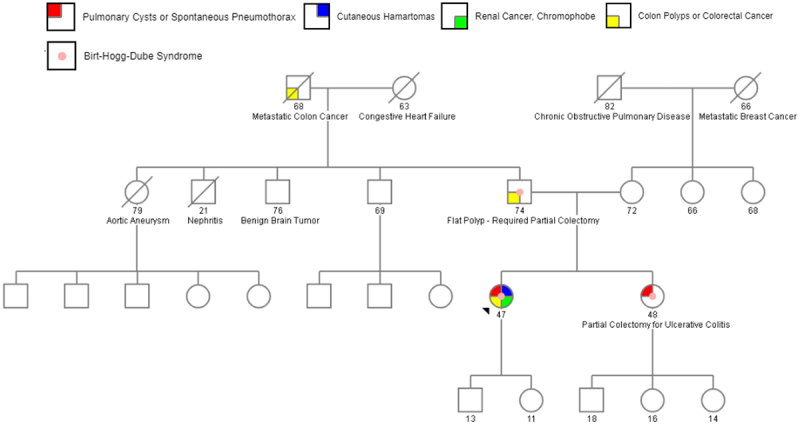
Three-generation pedigree of patient’s family.

Note: A three-generation pedigree indicates the index patient with an arrowhead in the context of her family. Relevant clinical history is noted for each family member, including partial colectomies in both her sister (for ulcerative colitis) and father (for a flat polyp). Grandfather was also noted to have colorectal cancer, but genetic testing was not performed to confirm suspected Birt–Hogg–Dubé syndrome as he was deceased. Created with Progeny Genetics.

Upon further review of her family history, multiple relevant familial risk factors were identified. Her paternal grandfather died from metastatic colon cancer at the age of 68. Her father had undergone partial colectomy after a flat polyp was discovered on colonoscopy, and her sister had previously experienced a spontaneous pneumothorax and underwent partial colectomy due to ulcerative colitis. There was no known family history of RCC. In this setting, the patient’s testing prompted genetic evaluation of her father and sister, revealing they too carried the same variant mutation in *FLCN*. A three-generation pedigree is detailed further in Figure 2.

Unfortunately, 23 months after her colon resection she was noted to have enlargement of a solitary liver lesion on surveillance CT scan, further evaluated with magnetic resonance imaging (Figure 1b). This was associated with an increased serum carcinoembryonic antigen. Although the hepatic lesion was previously believed to be a hemangioma and had been stable since it was first identified, liver biopsy confirmed metastatic adenocarcinoma consistent with colonic primary. Somatic genomic profile using Caris Molecular Intelligence® assay of the metastatic liver tumor further revealed the presence of the same pathogenic *FLCN* mutation as found in the saliva sample, c.1177–5_1177-3delCTC (Supplementary Table 2). She underwent right hepatectomy with curative intent for oligometastatic disease and completed adjuvant mFOLFOX6 with post-therapy scans confirming no evidence of disease.

## Discussion

BHD is a rare, autosomal dominant syndrome whose cutaneous manifestations were originally described in 1977 before further clinical features were identified over the subsequent three decades.^1–3^ It is characterized by three primary findings: cutaneous fibrofolliculomas; pulmonary cysts with spontaneous pneumothorax; and renal tumors of various subtypes including oncocytoma, chromophobe, papillary, and clear cell carcinomas.^4^

**Figure 3.**
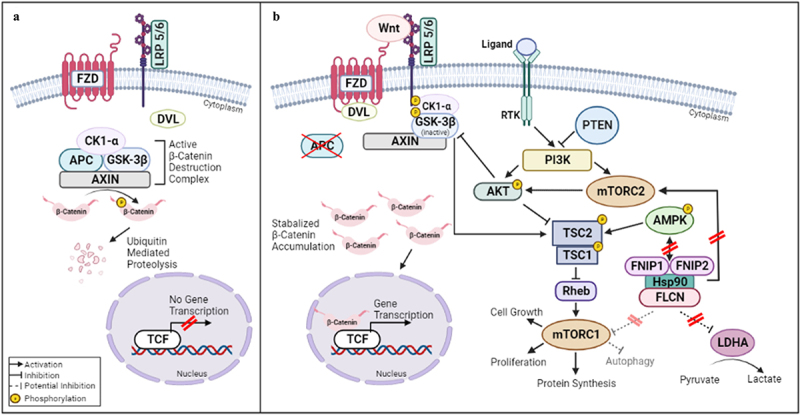
Wnt/APC/β-catenin signaling and interactions with folliculin.

Note: With functional *APC*, when the Wnt ligand is not present (Wnt OFF) the β-catenin destruction complex is active in the cytoplasm and prevents inappropriate downstream gene transcription and cell proliferation. (b) With mutated, non-functional*APC*, there is buildup of cytoplasmic β-catenin regardless of the presence of the Wnt ligand, resulting in nuclear gene transcription. Additionally, when the Wnt ligand is present (Wnt ON), there is further stimulation of cell growth processes mediated through the TSC1/TSC2 complex. This is modulated by interactions with AMPK and mTORC, and subsequently AKT, which experiences upstream regulation by folliculin. Folliculin is also a binding partner and uncompetitive inhibitor of LDHA. When *FLCN* is mutated, this creates an increase in lactate, which in turn drives aerobic glycolysis. AMPK – adenosine monophosphate-activated protein kinase; APC – adenomatous polyposis coli; AXIN – Anti-Neurexin; CK1-α – Casein Kinase 1 alpha; DVL – disheveled protein; FLCN – folliculin; FNIP1 – folliculin-interacting protein 1; FNIP2 – folliculin-interacting protein 2; GSK-3β – glycogen synthase kinase 3 beta; Hsp90 – heat shock protein-90; LDHA – lactate dehydrogenase A; LRP 5/6 – lipoprotein receptor 5/6; MET – c-mesenchymal-epithelial transition; mTORC1 – mammalian target of rapamycin complex 1; PI3K – Phosphatidylinositol-3-Kinase; PTEN – phosphate and tensin homolog; Rheb – Ras homolog enriched in brain; RTK – receptor tyrosine kinase; TSC1 – tuberous sclerosis proteins 1; TSC2 – tuberous sclerosis proteins 2; TCF – T-cell factor. Created with BioRender.

In 2002, a genetic basis for BHD was identified by Nickerson et al., implicating germline mutations in the *FLCN* gene on chromosome 17.^5^ *FLCN* is normally expressed in multiple tissues including the skin, kidney, lung, pancreas, and parotid, and its protein product has been associated with the regulation of cell growth and survival, metabolism, and cell adhesion.^6^ Folliculin interacts with heat shock protein-90 (Hsp90) and its binding partners folliculin-interacting proteins 1 and 2 (FNIP1 and FNIP2), appearing to operate downstream of AMP-dependent protein kinase (AMPK) and impact various signaling functions including ones reliant on AKT/mTOR signaling and the tumor suppressor TSC2. Folliculin has also been found to be an important binding partner and uncompetitive inhibitor of lactate dehydrogenase A (LDHA), and thus its absence leads to aerobic glycolysis otherwise known as the Warburg effect (Figure 3b).^6–11^ Although the exact mechanisms have yet to be elucidated, interruptions of these functions implicate dysregulation of folliculin functioning in the tumorigenesis of BHD.

As such, despite the rarity of the syndrome, its association with RCC has been well described in the medical literature. One study of 223 BHD family members showed that patients with BHD had a seven-fold greater risk for developing renal tumors than unaffected-related controls.^12^ There have also been infrequent reports of patients with BHD having other malignancies including thyroid cancer, parotid oncocytomas, and colorectal cancer (CRC), but none of these have been confirmed to be a part of the BHD phenotype.^13^

The association between BHD and CRC has been debated throughout the syndrome’s short history, as original observations reported a relationship between the two, but subsequent large-scale reviews have not been able to reproduce it. The same study of 223 BHD family members did not find a significant difference in the incidence of either premalignant polyps or cancers in affected individuals.^12^ A later study using similar methodology, including 399 BHD patients and 382 family members without *FLCN*, found a small but statistically insignificant higher rate of CRC in the affected patients (3.3% vs. 2.4%, *p* = .52).^14^ However, an investigation by Nahorski et al. found that patients with the *FLCN* exon 11 mutation c.1285dupC had a significantly higher risk of colorectal neoplasia when compared with the c.610delGCinsTA mutation.^15^ This may indicate the importance of specific genotypes having different neoplastic potential, noting the authors indicated that this finding was not consistent with the research of Toro et al. However, there is precedent for different mutations in *FLCN* inducing distinct effects on the function of folliculin. An *in vivo* murine model showed that two missense mutations, H255Y and K508R, have disparate malignant potential in kidney cells, lending further credence to the observation that some mutations may predispose to CRC while others do not.^16^

**Figure 4.**
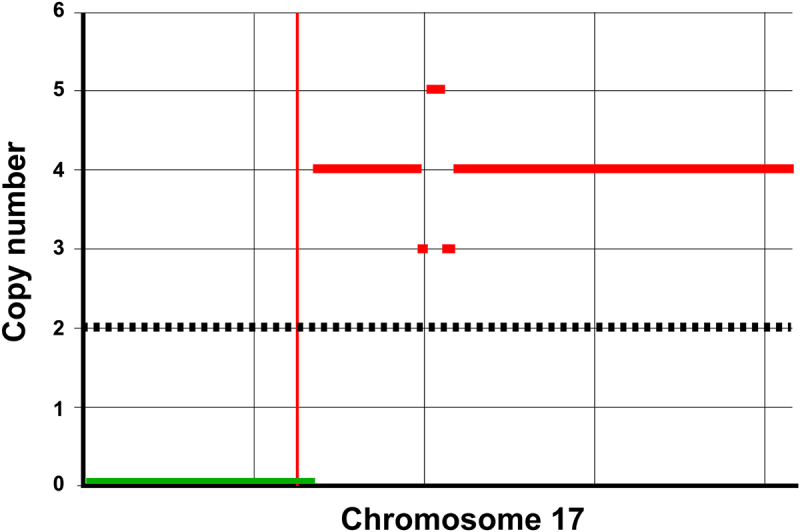
e-Karyotype of Chromosome 17 with locus-specific loss of heterozygosity.

Note: Karyotype visualization of cytogenetic aberrations across Chromosome 17 for the patient’s tumor. The copy number is a smoothed non-log representation of the estimated ploidy across arm-level parts of the genome. Vertical red line represents the chromosome centromere. P arm is located to the left of the red vertical line, and q arm to the right. Black dotted line represents estimated copy number of the chromosome with no copy number alterations. Green horizontal line represents estimated chromosomal loss within the patient’s tumor, and red horizontal lines represent varying amount of estimated chromosomal gain.

According to the Leiden Open Variation Database (LOVD), a publicly available online database that tracks the reported variants of the FLCN gene, there have been more than 300 unique mutations identified in the *FLCN* gene.^17^ Our patient was found to have the c.1177–5_1177-3delCTC mutation, a splice acceptor site mutation on exon 11 that causes the insertion of a premature stop codon, which subsequently yields a destabilized, truncated protein.^18^ Further analysis of the tumor revealed evidence of mono-allelic copy-number loss with an associated loss of heterozygosity (LOH), suggesting the wild-type allele was deleted (Figure 4). This finding was further supported by the diminished allele frequency of two known benign germline *FLCN* alterations that were also identified, c.1062 + 6C>T (variant allele frequency or VAF 5.3%) and c.397–14C>T (VAF 6.4%), suggesting these alterations were located on the lost allele. This LOH in BHD-associated CRC has not been widely described, but it implies that her mutation in *FLCN* was a driver (rather than a passenger) mutation, especially in the setting of microsatellite stability. Similar LOH in the *FLCN* gene has been observed in 17% of RCC patients with BHD.^19^ Together with her family history of colonic malignancies in both her father and paternal grandfather, this mutation was felt to most likely represent a meaningful genotype–phenotype association.

In addition to her *FLCN* mutation with LOH, our patient was found to have a somatic pathogenic mutation in *TP53* and an unclassified variant in *NCOR1*. Because somatic *TP53* mutations are common in CRC and considered a part of its pathogenesis, this was not necessarily surprising.^20^ However, the VAFs of all three mutations were particularly high; *NCOR1* (84%), *FLCN* (86%), and *TP53* (87%). Although this phenomenon was probably related to typical somatic losses of function in TP53 and NCOR1, it likely represents a locus-specific LOH event that resulted in high VAFs for these genes given their co-localization on chromosome 17p (Figure 4). In either case, only mutant TP53 proteins would be present, potentially acting as an additional driver for CRC tumorigenesis in this patient. It should also be noted that the patient was found to have two separate mutations in *APC*. As her germline testing did not reveal mutations in either *APC or TP53*, it was determined that she did not have the familial adenomatous polyposis (FAP) or Li-Fraumeni syndrome, and these genetic alterations were considered somatic. Detailed sequencing data can be found in Supplementary Tables 1 and 2.

It has been well established that the inactivation of *APC* is the major pathway for adenoma formation. Its inactivation causes an inability to degrade—and subsequent accumulation of—β-catenin (Figure 3a). This leads to deregulation of the Wnt/APC/β-catenin pathway and increased cell proliferation.^21^ While there has been no definitive connection made between the Wnt/APC/β-catenin pathway and the *FLCN, APC* mutations have also been proven to promote mTORC1 activation and result in the development of colon polyps. Constitutive activation of PI3K in the intestines has also been shown to be synergistic with the Wnt/APC/β-catenin pathway, which boosts tumor initiation and promotion via AKT signaling.^22^ To highlight the important therapeutic benefit of understanding the interplay between these pathways, a recent case report demonstrated improvement in the dermatologic manifestations of BHD after treatment with 1% rapamycin (an mTOR inhibitor) aided by laser assisted delivery of the drug.^23^ Extrapolating from such findings, should patients with BHD and CRC progress through available standard therapies, targeted treatment accounting for knowledge of the relevant pathways may reasonably be pursued.

BHD may predispose patients to colorectal cancer because of folliculin’s role in AMPK/AKT/mTOR signaling and its inhibition of LDHA (Figure 3b), synergizing with the more familiar APC pathway through established intermediaries of cell growth. Furthermore, LOH of the *APC* has been firmly established as a mechanism for CRC tumorigenesis in patients with FAP, and a similar loss of the sole wild-type *FLCN* gene in this case appears to contribute to our patient’s development of cancer by similar rationale.^24,25^ Taken together, we postulate that our patient’s *FLCN* mutation and resulting folliculin protein dysfunction favored unchecked cell proliferation through its role in regulating AMPK and downstream targets, and this was possibly potentiated by dual mutations in *APC*.

Currently, guidelines state that clinicians should consider initial screening colonoscopy in their BHD patients at 40 y of age, or 10 y prior to the onset of CRC in a first-degree relative.^26^ Although available evidence indicates that earlier screening for CRC is unnecessary in the majority of BHD patients, there may be certain *FLCN* mutations more commonly associated with CRC. Thus, if a patient’s genotype is known to have this association, such as the previously described c.1285dupC or the c.1177–5_1177-3delCTC as illustrated in this case, earlier screening may indeed be prudent.

## Conclusion

In this case, we discussed the first BHD patient to our knowledge with the c.1177–5_1177-3delCTC *FLCN* mutation and associated CRC. While BHD’s association with CRC remains debated, the mutational landscape of the *FLCN* gene is becoming clearer, and there may be specific genotypes that carry risks that others do not. The interactions between the Wnt/APC/β-catenin and AKT/mTORC1 pathways may be of particular interest in BHD patients, as this could both elucidate pathophysiology and determine appropriate therapeutic targets. Further investigation with larger cohort studies will be important to clarify the relationships between mutations in these pathways and their attendant risks.

## Supplementary Material

Supplemental Material

## Data Availability

Data sharing is not applicable to this article as no new data were created or analyzed in this study.
