# Supplementary material for: Colon adenocarcinoma and Birt–Hogg–Dubé syndrome in a young patient: case report and exploration of pathologic implications
Source: Cancer Biol Ther. 2023 Mar 1;24(1):2184153. doi: 10.1080/15384047.2023.2184153 (PMC9988342; doi:10.1080/15384047.2023.2184153)
Supplement: Supplemental Material [file KCBT_A_2184153_SM4193.docx]

**Colon adenocarcinoma and Birt-Hogg-Dubé syndrome in a young patient: Case report and exploration of pathologic implications**

**Supplementary materials:**

Table 1: Germline Sequencing Result (Saliva sample)

| Gene | Variant | Zygosity | Variant Classification |
| --- | --- | --- | --- |
| FLCN | c.1177-5_1177-3del (intronic) | Heterozygous | PATHOGENIC |

Table 2: Somatic tumor sequencing data

| Gene | Analyte | Variant | Exon | DNA Alteration | Variant Frequency, % |
| --- | --- | --- | --- | --- | --- |
| APC | DNA-Tumor  DNA-Tumor | Pathogenic  Pathogenic | 6  16 | c.636dupA  c.4132C>T | 68  16 |
| FLCN | DNA-tumor  DNA-tumor  DNA-tumor | Pathogenic  Silent  Silent | 11  9  6 | c.1177-5_1177-3delCTC  c.1062+6C>T  c.397-14C>T | 86  5.3  6.4 |
| TP53 | DNA-tumor | Pathogenic | 8 | c.844C>T | 87 |
| NCOR1 | DNA-tumor | Unclassified | 25 | c.3337C>T | 84 |
